# Abrasive Waterjet (AWJ) Forces—Indicator of Cutting System Malfunction

**DOI:** 10.3390/ma14071683

**Published:** 2021-03-29

**Authors:** Libor M. Hlaváč, Damian Bańkowski, Daniel Krajcarz, Adam Štefek, Martin Tyč, Piotr Młynarczyk

**Affiliations:** 1 Department of Physics, Faculty of Electrical Engineering and Computer Science, VSB–Technical University of Ostrava, 17. Listopadu 2172/15, 70800 Ostrava-Poruba, Czech Republic; adam.stefek.st@vsb.cz (A.Š.); martin.tyc.st@vsb.cz (M.T.); 2 Department of Materials Science and Materials Technology, Faculty of Mechatronics and Mechanical Engineering, Kielce University of Technology, al. Tysiąclecia Państwa Polskiego 7, 25-314 Kielce, Poland; dbankowski@tu.kielce.pl (D.B.); d.krajcarz@wp.pl (D.K.); piotrm@tu.kielce.pl (P.M.)

**Keywords:** metals, abrasive waterjet, force measurement, cutting, force ratio

## Abstract

Measurements enabling the online monitoring of the abrasive waterjet (AWJ) cutting process are still under development. This paper presents an experimental method which can be applicable for the evaluation of the AWJ cutting quality through the measurement of forces during the cutting process. The force measuring device developed and patented by our team has been used for measurement on several metal materials. The results show the dependence of the cutting to deformation force ratio on the relative traverse speed. Thus, the force data may help with a better understanding the interaction between the abrasive jet and the material, simultaneously impacting the improvement of both the theoretical and empirical models. The advanced models could substantially improve the selection of suitable parameters for AWJ cutting, milling or turning with the desired quality of product at the end of the process. Nevertheless, it is also presented that force measurements may detect some undesired effects, e.g., not fully penetrated material and/or some product distortions. In the case of a proper designing of the measuring device, the force measurement can be applied in the online monitoring of the cutting process and its continuous control.

## 1. Introduction

The machining tool “abrasive waterjet” (AWJ) has been used as a modern machining technology for about 40 years. It is either applied or tested for use in cutting [1], milling [2], turning [3], piercing [4], grinding [5] or polishing [6]. AWJ is capable of machining materials independently of their thickness, provided that sufficient energy is supplied [7]. The regression or theoretical models are utilized to determine accuracy of this tool. No complex model applicable for all types of materials and machining parameters has been prepared yet. Therefore, models used in practice are suitable for either ductile materials or for brittle ones. A better understanding of the erosion process is essential for the improvement of the contemporary models or the preparation of some new and better ones. It can even aim for the creation of some new design of the AWJ tools. Many online and offline measurements have been made in past to gain information about quality of the AWJ machining process. However, conventional online methods are often inappropriate, because the environment is very unsuitable for electrical equipment, optics and other common sensors during the machining process. The flexibility of the AWJ makes it strongly sensitive on all irregularities in jet flow and/or material properties. Rebounding of the abrasive water jet from the material or deflection of the jet into an unexpected direction increases the risk of tracking technique damage. For the further expansion of AWJ applications in industry, there needs to be an improvement of measuring processes [8]. Therefore, some new methods suitable for monitoring of “new technologies” have been studied and tested, as it was mentioned in [9].

Some new measuring methods were tested for the monitoring of the abrasive waterjet in milling, turning and 3D machining. The investigation of the acoustic emissions [10,11] is one of such unconventional methods used for AWJ measuring. Several attempts aimed at determining the quality of the machining process through the measurement of the cutting head vibrations were presented few years ago [12,13,14]. Vibrations of the machined material are often studied to find some new method applicable for AWJ process monitoring [15,16,17]. Some of the scientists have found, however, that more than 25% of the information from these sources may be incorrectly explained [18] due to misinterpretation of signal characteristics. These signals (acoustic emissions, cutting head vibrations, material vibrations) may be strongly influenced by the dimensions of the machine, its stiffness or the dimensions of the machined material. Force sensors were used in the past, namely for the determination either of the velocity profile [19,20] of the pure waterjet or some measurements of the waterjet and abrasive waterjet diameter [19,20,21]. The force measurements were also used for the investigation of pulsating jets [22]. The first application of force measurements on AWJ force measurements has been described in [23]. Another presented force measurement investigation was aimed at studying the mixing processes [24]. The more recent use of waterjet force measurements is focussed on hand tools [25], impact force at high pressures [26] and the impact of the waterjet structure on forces [27,28]. AWJ force measurements are once again coming to the fore [29,30], especially in connection with applications on new materials.

The force measurements described in this contribution to the knowledge of the AWJ are focused on the static force component, eliminating the dynamic component with questionable origins. Supressing or even the elimination of the measured signal distortion caused by the effects mentioned above was the most important motivation for this study aimed at force measurements on samples with equal dimensions. The applied force sensor measuring x-y-z forces during the AWJ cutting process was designed and manufactured by our research group in 2011 and patented in 2012 [31]. Since that time, it has been used for the investigation of the impact of the AWJ on materials. The first remarks about force measurements performed with our sensor were presented at a conference in 2019 [32]. Research of the AWJ force’s dependence on the material structure was presented soon after [33]. Some recent findings are presented here, e.g., the application of the sensor for testing of the assumption regarding linkage between the ratio of forces in x and z orthogonal axes and the ratio of cutting to deformation wear (i.e., respective tangential and normal forces). The set of force measurements performed on metal samples is presented, evaluated and discussed in this article. As traverse speed is one of the key factors affecting force values, the different traverse speeds were applied testing the basic assumptions. Many similar experiments were performed, and some assumptions were verified. However, the results showing some specific and unusual features were also selected for presentation and discussion in this article primarily.

## 2. Theoretical Background

One of the theoretical models presented by Hlaváč [34] is based on the cutting quality determination through the ratio of traverse speed to the limit one. Nowadays, the declination angle of striations measured at the bottom edge of a sample wall is used for cutting quality evaluation, as was presented in [35,36]. The specific factors introduced in these previous articles are namely the limit traverse speed and the limit declination angle. These factors can be utilized, as was presented in previous publications, for the determination of cutting characteristics ensuring the required quality of the cutting wall. As the traverse speed of the cutting head is the most easily changeable cutting factor, it is the most usual process variable used for quality control of the AWJ machining. Therefore, the most typical task of the AWJ operator is to set up an appropriate traverse speed, ensuring the desired quality of a product. An example of complex knowledge application, based on the theoretical description of an AWJ cutting through the declination angle and the taper, was demonstrated in [37], comparing results of the theoretical model and experimental investigation on columns cut from various metal samples. The simplified Hlaváč’s model for limit traverse speed calculation can be expressed as Equation (1):

(1) $$v_{P\lim}\;=\;{\left[\frac{C\;d_{o}\;\sqrt{2\rho_{j}p_{j}^{3}\;e^{-5\xi_{j}L}}\;(1\;-\;\alpha_{e}^{2})}{8\;H\;\left(p_{j}\rho_{m}\alpha_{e}^{2}\;e^{-2\xi_{j}L}\;+\;\sigma_{m}\rho_{j}\right)}\right]}^{\frac{2}{3}}\;-\;v_{Pmin}$$

The meaning of the variables in Equation (1) are as follows: $v_{Plim}$: the limit traverse speed; C: coefficient including influence of abrasive mass flow rate, abrasive material quality and Ludolf’s number (for circular jet shapes); $d_{o}$: diameter of the water nozzle (orifice); $\rho_{j}$: density of the abrasive jet (conversion to a homogeneous liquid); $p_{j}$: pressure obtained from Bernoulli’s equation for *liquid* with density and velocity of *abrasive jet*; $\xi_{j}$: attenuation coefficient of the abrasive jet in the environment between the focusing tube outlet and the material surface; L: stand-off distance (distance between the focusing tube outlet and the material surface); $\alpha_{e}$: coefficient of the abrasive water jet velocity loss in the interaction with the material (experimentally determined from a testing cut on a sample of the respective material); H: material thickness; $\rho_{m}$: density of the material being machined; $\sigma_{m}$: strength of the material being machined; $v_{Pmin}$: minimum limit traverse speed of cutting-correction for the zero traverse speed (usually the value $v_{Pmin}=a_{n}/60$ is used, where $a_{n}$ is the average abrasive particle size after the mixing process inside the mixing head and focussing tube).

The calculation of the limit traverse speed from the theoretical model is limited by properly determining all necessary inputs. However, some of these inputs are rarely known and, sometimes, are even hardly determinable. Therefore, Equation (2), being just a modification of an equation describing the relation between traverse speed and declination angle presented in [34], is often used:

(2) $$v_{Plim}=v_{P}{\left(\frac{\vartheta_{lim}}{\vartheta }\right)}^{\frac{2}{3}}$$

where $v_{P}$ is the traverse speed value for which the declination angle ϑ is measured on the kerf wall; the limit traverse speed $v_{Plim}$ and the limit declination angle $\vartheta_{lim}$ are the limit values experimentally determined for selected material and cutting conditions.

The limit declination angle $\vartheta_{lim}$ changes with the machine power and material resistance to machining by the AWJ. For sufficient power and material thickness up to 50 mm, the angle is approximately 45°. Increasing the material thickness or decreasing the AWJ power causes the deterioration of conditions necessary for the full development of the deformation removal mode in the material. The limit declination angle decreases to the value of 15°, valid for a single cutting wear mode inside very thick materials (or for powerless machines). The opposite situation is when the material is very thin—such a material could be easily perforated due to the deformation mode and the limit declination angle seems to be over 50°. The best sample thickness used for force measurement investigation seems to be within 5 and 25 mm. The samples thicker than 25 mm are usually heavy and the force sensor range in the impact force is reduced through loading by the gravity force caused by the sample mass.

The theoretical knowledge presented in Equations (1) and (2) can be easily used for the elimination of the time-consuming experimental determination of limit traverse speeds for materials for which such a value has been determined on a certain machine. The equation for limit traverse speed recalculation can be derived namely in cases, when only a few parameters are changed, e.g., pressure, nozzle diameter and abrasive mass flow rate. This method was used in experimental work presented in this article, determining Equation (3):

(3) $$v_{Plim2}=\eta_{A21}\frac{d_{2}}{d_{1}}\sqrt{\frac{p_{2}^{3}}{p_{1}^{3}}}v_{Plim1}$$

Variable $v_{Plim2}$ is the limit traverse speed calculated for machine configuration 2; $v_{Plim1}$ is the limit traverse speed already known for machine configuration 1; $\eta_{A21}$ is a ratio of abrasive qualities (the second to the first one), if they differ; $d_{1},\;d_{2}$ are the respective nozzle diameters of configurations; $p_{1,\;}p_{2}$ are the respective pumping pressures of configurations.

This important result derived from the theoretical model has been used for experimental planning, because the original limit values were determined for parameters of the machine at the VSB—Technical University of Ostrava (Ostrava, Czech Republic), but measurements were performed on machine with different parameters at the Kielce University of Technology (Kielce, Poland).

**Figure 1:**
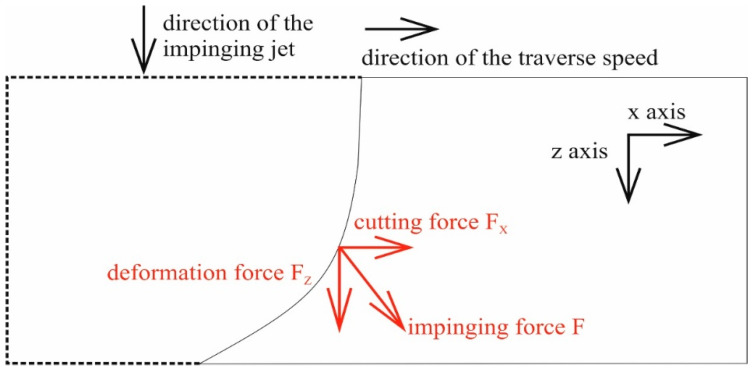
Scheme of the force decomposition in a certain point on the heading line characterizing the trajectory of the penetrating jet (the perpendicular direction to the traverse speed and gravity is not analysed in this study, so the impinging force is reduced into two dimensions).

The traverse speed of the cutting head is the most appropriate variable for studies of relations between the force measurement and the cutting process quality, as it can be easily changed and very precisely set up. The cutting (tangential to the sample surface) and the deformation (normal to the sample surface) forces were defined as indicated in Figure 1.

The consideration of the sketched force decomposition (Figure 1) leads to the presumption postulating this statement: “The cutting to deformation force ratio (CDFR) depends on the ratio of the cutting-to-deformation wear inside the produced kerf”. Based on the experimental results, it is assumed that increasing the traverse speed from the zero value induces the respective increase in the CDFR. The maximum of the CDFR is expected to be around half of the limit traverse speed. Increasing the traverse speed above the value corresponding to the maximum of the CDFR leads to the decrease in the CDFR value. However, this consideration is valid only in cases when the energy of the jet is sufficient for the full development of both types of wear. If the jet energy is lower than necessary (when cutting very thick materials), only the cutting wear provides penetration through the material. The residual jet energy inside the deep kerfs is insufficient for the proper development of the deformation wear. Therefore, when the conditions (namely traverse speed) are changed so that cutting force itself is insufficient for material removal, the residual jet reflects back from the kerf. This situation is typical for an outlet declination angle overcoming a value of about 22.5° for medium-thick materials (1.5–2.5 thicker than is appropriate for the jet energy) or 15° for very thick materials (more than 2.5 times thicker than is appropriate for the jet energy). A similar situation can also be caused by the decrease in the AWJ power (insufficient pumping system or pump failure).

## 3. Measuring System Description

**Figure 2:**
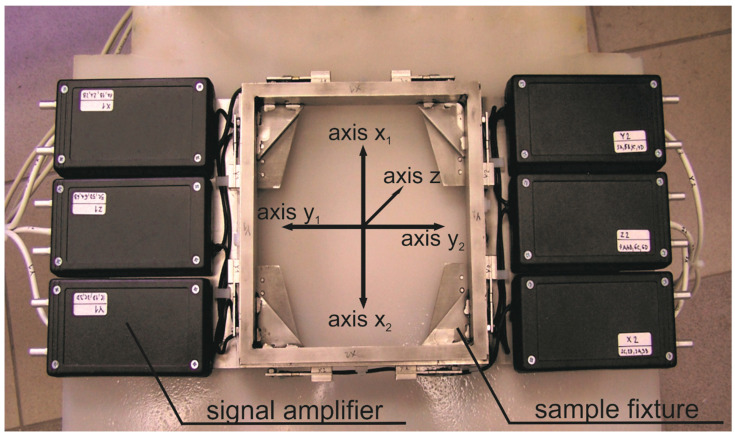
Force sensor designed and constructed for abrasive waterjet (AWJ) force measurements (amplifiers for strain gauges are closed in black boxes being blown through by pressurized air to prevent moisture).

A device measuring forces in three orthogonal axes has been developed, constructed and patented by our team [31]. The design is shown in Figure 2.

The measuring system consists of twelve deformation elements, each one covered by two extensometers. The six electric signals are amplified by amplifiers closed in plastic boxes (Figure 2), each one for a full Wheatstone bridge composed of four extensometers on two deformation elements in the appropriate direction. The amplifiers are supplied by an external direct current source 15 V. The amplified signal enters the analog-to-digital transducer and hence a computer. Recording and processing of the signal is performed using the software Signal Express (2012) and LabVIEW (2012), both from the National Instruments Corp., Austin, TX, USA. All six signals are recorded via a program prepared in the Signal Express, saving the time needed for individual measurements. The raw voltage signals are processed in the LabVIEW program in any subsequent unlimited time. This handling of the signal is used during the research state. However, the continuous evaluation necessary for online control can be easily obtained by modifying either the Signal Express program or the LabVIEW one.

## 4. Experimental Setup, Presumptions and Results

All presented measurements were performed in the Faculty of Mechatronics and Mechanical Engineering, Department of Materials Science and Materials Technology at the Kielce University of Technology, Kielce, Poland. This international cooperation was used to test forces on a less powerful and smaller cutting machine than the one used in Ostrava (VSB—Technical University of Ostrava, Ostrava, Czech Republic). The experimental factors and applied settings of the abrasive waterjet are shown in Table 1.

Several squared metal samples presented in Table 2 and Table 3 were used for the experiments. Their uniaxial tensile strength (*σ_m_*), density (*ρ_m_*) and Vickers hardness (HV10) are summarized in Table 2. All presented samples were plates with dimensions of 120 × 60 × T in mm, where T is thickness. The linear cuts perpendicular to the longer side were performed around the middle of this side and they extended approximately 20 mm into the sample. The samples were fixed to the base plate of the sensor by two steel strips pressed onto the material by nuts on screws approximately in 1/6 and 5/6 of the sample length. The base plate with dimensions of 170 × 170 × 3 in mm is made of low alloy high strength steel and it has a central circular hole with a diameter of 80 mm. This base plate is input to the frame named “sample fixture” in Figure 2 and fastened with a quick release. The tested samples were positioned so that the entire path of the abrasive jet lay inside the hole of the base plate. The unsupported area of the sample (due to the hole in the base plate) did not exceed 40% of the sample area. The jet path started approximately 3 mm outside the material edge. All cuts were performed in an x-axis direction. Therefore, y-axis forces (side forces in kerfs) were negligible compared to those in x and z directions. These signals caused by cutting were of the order 10^−2^ V in y direction, while signals in x and z directions were of the order of volts. Therefore, the signal from the y direction was not included in the analyses.

Beside the basic metal set also used in past experiments [37], two additional sets of samples were prepared of 34CrMo4 steel (DIN norm), each one with a different nickel ratio. These samples were austenitized at 850 °C, quenched in polymer and tempered at various temperatures; their tempering and respective uniaxial strengths are presented in Table 3. Samples prepared of the mentioned two steel modifications with identical heat treatment were also used for research aimed at the steel structure impact on AWJ machining presented in [38]. The sampling frequency used for force signal records was 20 kHz. This frequency yields the possibility of studying characteristic frequencies of signals up to 10 kHz, but there are rarely any significant frequencies higher than 5 kHz in our recorded signals.

**Figure 3:**
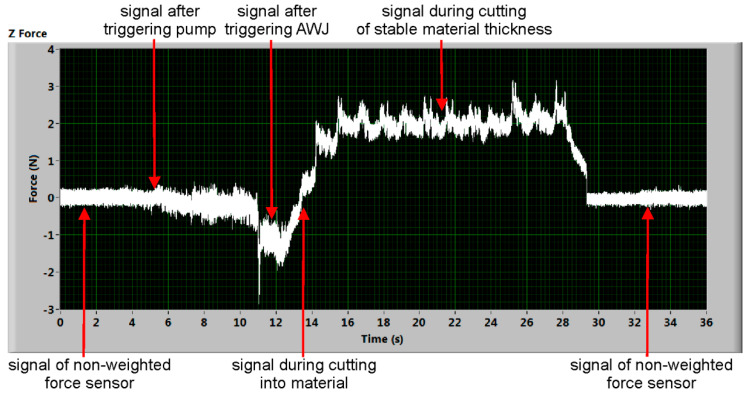
Shape of the typical measured signal in the z axis with description.

In order to prove the presumption that increasing the traverse speed leads to the increase in the CDFR up to maximum value with a subsequent decreasing, three cuts were carried out on each one sample. Making a particular cut, the traverse speed of the cutting head was increased. Different speed sets were used for individual material types to retain a similar traverse speed ratio regarding the limit traverse speed for each tested material. The lowest traverse speeds correspond with a very high quality of cutting, producing small declination angles of striations on the cut walls—about 6° (up to 10°). The medium traverse speeds correspond with often used quality in practice being a compromise between production speed and economy—the declination angles of striations on the cut walls are between 15° and 25°. The highest traverse speeds correspond with a poor quality of cutting for large declination angles of striations on the cut walls, typically over 30°. The force sensor is quite sensitive to accidental excitements caused, for example, by imperfections of the cutting table. Therefore, it was impossible to arrange the same conditions for all measurements performed on different days or after a break due to different work on the equipment. However, the observed shifts in absolute values (caused by different fixing of the measuring device or samples) are of the corresponding size in all axes. Therefore, the cutting to deformation force ratio (ratio of average values in axes x and z; see Figure 2) was analysed. The typical time dependent force signal record is presented in Figure 3. It shows the almost ideal shape of force signal in the z axis.

The processing procedure starts with the transformation of the voltage signal to the force signal through calibration equations prepared for each couple of deformation elements representing one Wheatstone bridge (calibration was performed in a static mode; the increasing and decreasing force was realized by adding or removing weights). Simultaneously, the signal level at the beginning of the measurement is moved to a zero level according to the auto-calibration signal sample selected from the signal without the switched waterjet machine. Both of these steps are performed in the program automatically for each of the measured signals, but the operator can change the selection of the signal segment used for calibration. The basic task during signal processing is to define the appropriate part of the signal after triggering the AWJ to set the lowest level of the force and to select the “stable” part of the signal during AWJ cutting of the material for setting the higher level of the force. Both parts of the signal are selected manually according to the respective timeline (Figure 3). The median values of each level are counted and subtracted from each other. The difference of these median values is assumed to be the force value in the respective direction (axis). The principle is the same in both considered axes representing the cutting and deformation force (x and z axis, respectively). The ratio of the x and z force values is determined directly in the prepared LabVIEW program for each material, for which the relevant records were obtained. Signals from both axes are measured simultaneously. The set of force ratio values and respective relative traverse speeds is summarized in Table 4.

## 5. Discussion of Results

**Figure 4:**
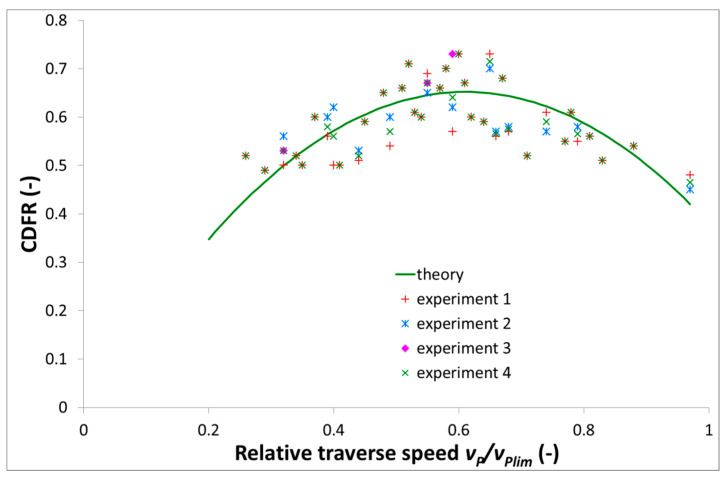
Relationship between the cutting to deformation force ratio (CDFR) values and the relative traverse speed values.

All measurements indicate the fact that with increasing traverse speed, the CDFR increases, firstly up to a certain maximum value and then decreases. This result can also be seen in Figure 4, showing the CDFR dependence on the relative traverse speed. Therefore, it can be said that the measurements prove this presumption well. The principle of the measuring device is based on the deformation of the metal deformation elements. However, these deformation elements are not infinitely tough, and the calibration equations do not include the hysteresis behaviour fully (it is also dependent on the fixing of the device, sample weight, etc.). The irremovable hysteresis makes it impossible to compare absolute values of measurements performed at different times and in different conditions. Opposite to this, the ratio of the forces (deformation to cutting force) seems to be independent on the fixing conditions and changes, because shifts caused by the fixing of the sample weight influence all axes (directions of measurement) proportionally. The calculated combined uncertainty of measurements is approximately 8%, including the uncertainty of the device measuring electric signal, variability of material properties and stability of cutting parameters. Therefore, the CDFR is a suitable quantity for comparison.

The force value is dependent on the defined signal on the timeline. Selected sections might cause different counted force values. To eliminate this potential distortion, the median values of each level are counted and each of the selected timeline levels should be as long as possible. The signal level median values are resistant to outliers caused by improper selection. The “theoretical” curve in Figure 4 was determined on the assumption that the dependence of the force ratio on the relative traverse speed is parabolic. The necessary constants for the mathematical expression of the curve were determined from the regression of the results. The resulting equation used for the calculation of the curve in the graph presented in Figure 4 as “theory” has this form:

(4) $$\mathrm{CDFR}=-1.8v_{r}^{2}+2.2v_{r}-0.02$$

The ratio of actual traverse speed $v_{P}$ to the limit one $v_{Plim}$, i.e. ($v_{P}/v_{Plim}$), is signed $v_{r}$ (relative traverse speed) in Equation (4).

**Figure 5:**
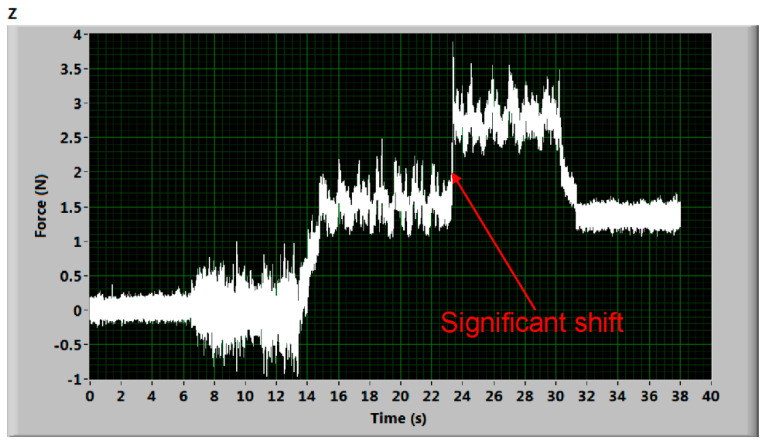
Deformation force signal with a significant shift during the stable part of cutting (sample of steel 1.4541, traverse speed 100 mm/min).

**Figure 6:**
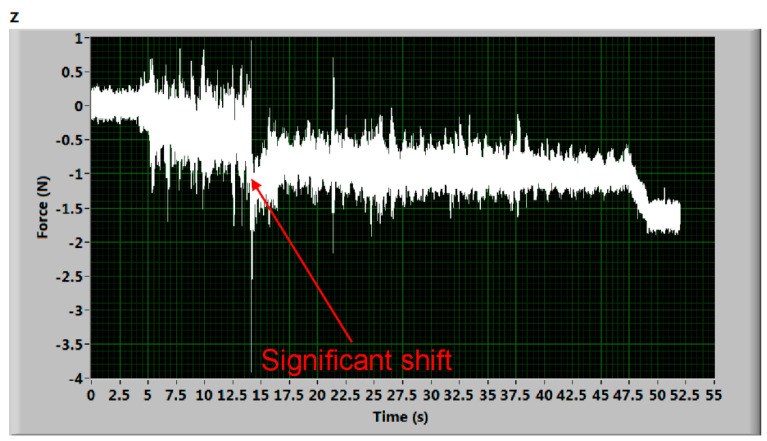
Deformation force signal with a significant shift during the beginning part of cutting (sample Hardox 500 plate, traverse speed 50 mm/min).

**Figure 7:**
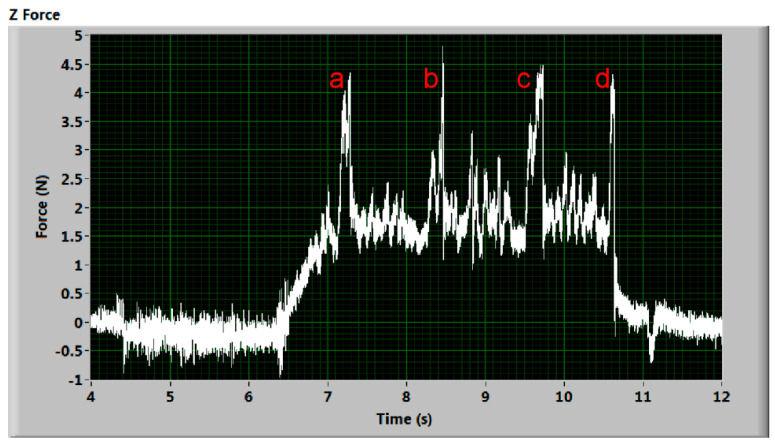
Deformation force signal with presumed uncut parts—normal force increases on the uncut material parts (duralumin 2017, traverse speed 400 mm/min). Peaks (a, b, c, d) correspond with increase of deformation force on uncut parts (Figure 9), where jet rebounds.

**Figure 8:**
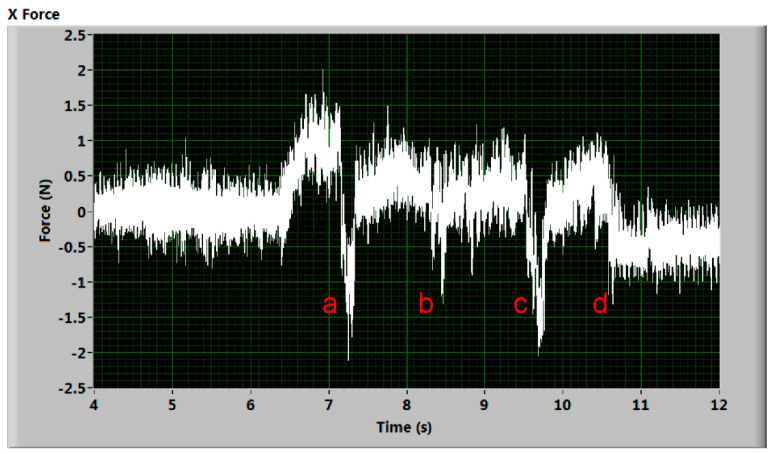
Cutting force signal with presumed uncut parts—tangential force decreases on the uncut material parts (duralumin 2017, traverse speed 400 mm/min). Peaks (a, b, c, d) indicate opposite direction of cutting force on the uncut parts (Figure 9) induced at the end of them.

The problem is a limited number of possible measurements. The number is not sufficient for quality statistical conclusions. Moreover, some of the force ratio values can be influenced by changed cutting conditions of similar measurements performed earlier, such as another cutting table, another pumping device, etc. All these changes influence the stiffness of the respective cutting device and the corresponding fixing possibilities. Beside these sources of systematic uncertainties shifting signals in all axes, some other irregularities occurred in some of the measured signals. Several unusual signals are presented in Figure 5, Figure 6, Figure 7 and Figure 8 and are commented on in further text. They may bring some new information about AWJ cutting. The sources of the unusualness are the subject of the contemporary and future research.

Figure 5 shows a significant shift during AWJ cutting. This shift may be justified in higher levels of the water in the AWJ residual energy attenuator. Splashing water can induce additional deformation of the deformation elements and suddenly shift the measuring level. Generally, this seems to be caused by a quick mechanical impact on the AWJ cutting process. Hysteresis is well visible when comparing the beginning and the final levels of the signal. Nevertheless, two almost identical values can be evaluated taking the first force “step” between the level of signal from 8 to 13 s and the one from 15 to 23 s and the second force “step” calculated from signals taken from timespans from 24 to 30 s and from 32 to 37 s. Analogically, Figure 6 shows some kind of interruption at the beginning of the measurement (before the cut in the material). No force value can be determined directly. However, comparing the signal level from the time interval from 17 to 46 s and signal level in timespan from 49 to 51 s, certain force values can be calculated again, similar to the case presented in Figure 5. Nevertheless, these “damaged” records were not used in the analyses of the presented force ratio dependence on relative traverse speed. They are presented just as examples of the potential problems of force measurements.

Figure 7 and Figure 8 show the signal, where the evident increase or decrease in the signal during the cutting stage (see part of the signal defined in Figure 3) is recorded. The process of this phenomenon is not as sudden as in the signal mentioned above. Some explanation could be the presence of supporting slats underneath the workpiece or improper abrasive delivery. Nevertheless, the measuring device was intentionally placed into positions at which no rebounding of the jet on supporting parts of the cutting table could take place. The delivery of the abrasive was continuously monitored, as well as the pumping pressure and the cutting device motion. All experiments with observed problems with the machine equipment were stopped and repeated. The presented results are observed in measurements with no visible problems except the case presented in the last figure of this article—the force record when the pump seal was broken.

**Figure 9:**
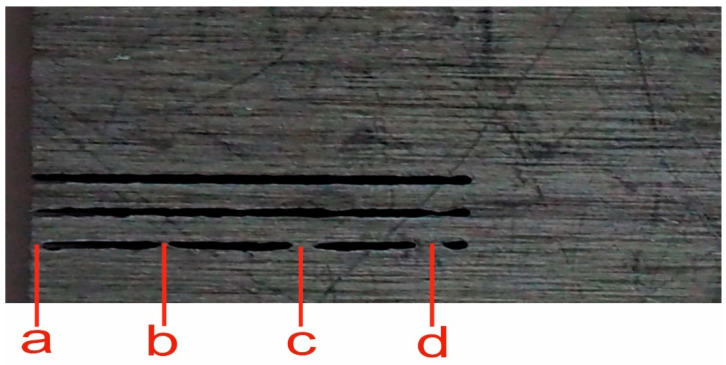
Bottom side of the sample with marked uncut parts causing significant force changes (duralumin 2017, traverse speed 400 mm/min). The uncut parts of material marked a, b, c, d are corresponding with peaks marked with the same letters in the signal records in Figure 7 and Figure 8.

The explanation of the peaks marked a, b, c, d in signals presented in Figure 7 and Figure 8 is based on the photo of the sample after the AWJ cut (Figure 9). There are several visible points where the AWJ does not cut through material. They are marked by identical letters (a, b, c, d) as the marks put into graphs of both significant signals (Figure 7 and Figure 8). Peaks marked (a, b, c, d) in Figure 7 correspond with increase of deformation force on uncut parts, where jet rebounds. In Figure 8 the respective peaks (a, b, c, d) indicate opposite direction of cutting force on the uncut parts induced at the end of them. The zero level in x-axis is gradually lowered due to hysteresis of the measuring system.

The positions of the marks in the graphs were calculated from the jet traverse speed and the initial position of the cutting head regarding the material sample. The reflection of fluid flow on the uncut parts of material causes the rapid and short increase in the impact force up to twice the value of the cutting condition. This increase is consistent with the theory of liquid flow impact on a solid-state obstacle.

**Figure 10:**
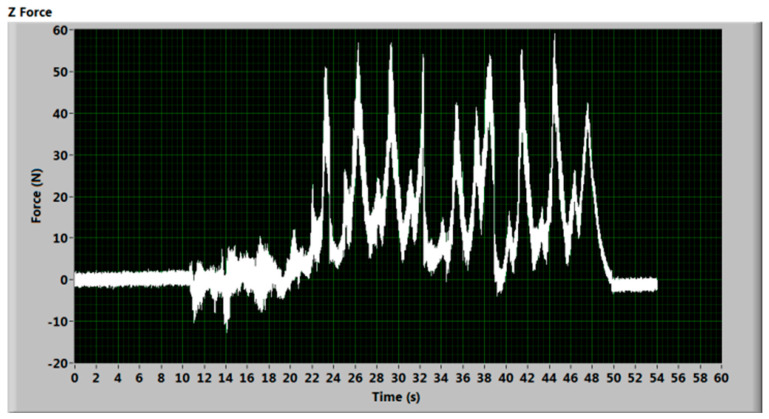
Cutting force signal from cutting with a pump with a damaged seal in one side of the multiplier; the cut material was steel 1.0547 (St 52-3), traverse speed 50 mm/min.

The malfunction of the pumping machine also occurred during measurements and it was recorded and evaluated. The resulting time dependent force signal can be seen in Figure 10. The impacting force copies strokes of the machine and the force peaks are much higher due to the reflection of the abrasive water flow from a fixed obstacle—the kerf bottom. The work of the pump significantly contributes to the peak formation, as can be seen in the signal shape. The pressure changes caused by the change of the piston movement direction in the multiplier can be seen on the record. The small peaks between the high ones are the strokes of the multiplier side with leaking seals. This side fails to achieve the necessary pressure. The highly fluctuating pressure level does not reach the required value for the proper suction of the abrasive material, causing the resulting increase in force to values several times higher than the ones observed for the abrasive jet with the proper pressure, because almost pure water is impinging the sensor.

The record presented in Figure 10 was obtained during the preparation of the new set of experiments. When the problem of the pump was observed, a smaller orifice (0.25 mm) and focusing tube (0.76 mm) were used to be sure that an even lower flow rate could not improve the situation. The approximate maximum observed pressure was 150 MPa and the focusing tube diameter was 0.76 mm (approximate diameter of impacting jet), and the yield force value was 68 N and the observed peaks in Figure 10 are mostly between 50 and 60 N (i.e., between 73 and 88% of the maximum value calculated for observed pressure). Considering that a small amount of abrasive was still sucked, it can be concluded that the measured result correlates with observed physical reality quite well. However, it was just one measurement and immediately after it, the work was stopped to prevent further damage of the machine.

Some new experiments have been suggested for proving the hinted hypotheses and mapping the sensor behaviour properly:

- the force measurement of intentionally made uncut parts (setting up the particular AWJ parameters so that the jet has problems with cutting through a sample);
- a similar experiment set-up as presented in this article, but with the traverse speeds inducing a significant increase in the signal level (to reduce uncertainty from noise generated from servomotors);
- the measurement of forces on machines with different types of engines used for axes movement;
- force measurement showing the influence of the water level in the AWJ attenuator onto signal shape and value.

## 6. Conclusions

This paper presents results obtained with a special force sensor during AWJ cutting of metals. The sensor measures force signals in three orthogonal axes. The experiments make evident that the online force measurements can be used for the evaluation of the cutting quality provided that the ratio of values is analysed. The results of measurements should also contribute to the understanding of the machining process. The results from the previously presented research were confirmed on a broader scale of materials. These results confirm that for usual conditions, i.e., energy of jet is sufficient for the development of both wear types—cutting and deformation ones, the increase in the traverse speed leads to an increase in the cutting to deformation force ratio up to a certain maximum at about half of the limit traverse speed, and then further increase in the traverse speed leads to a decrease in the cutting to deformation force ratio. Measurements prove this presumption very soundly. Nevertheless, some of the measurements were distorted by a higher level of noise. This noise is caused namely by the impact of the rippling water in the waste vessel (jet energy attenuator). Other signals are mechanically interrupted during the cutting process. This problem can be caused by a high level of water in the AWJ energy attenuator, insufficient sample fixing and/or sensor hysteresis. The damaged signals were not included into force analyses, but they are presented as examples and discussed. The detailed investigation of this problem is just in process. Some signal shapes that look different from the typical one indicate a problem with cutting through the material, either due to a pumping machine problem or improper traverse speed setting. These phenomena are to be subjected to a broad intentional investigation in the near future.

## Figures and Tables

**Table 1:** Summary of factors and settings in experiments.

Variable (Unit)	Value
Pump pressure (MPa)	250
Water orifice diameter (mm)	0.33
Focusing tube diameter (mm)	1.02
Focusing tube length (mm)	76
Abrasive mass flow rate (g/min)	240
Abrasive material average grain size (mm)	0.177
Abrasive material type	Indian garnet
Stand-off distance (mm)	2
Traverse speed (mm/min)	50–400

**Table 2:** Materials used in experiments (10 mm thick). *σ_m_*: uniaxial tensile strength; *ρ_m_*: density; HV10: Vickers hardness.

WRN (DIN) Norm	*σ_m_*	*ρ_m_*	HV10
	(MPa)	(kg·m^−3^)	
1.0547 (St 52-3) construction steel	445	7772	125
1.0503 (C 45) low alloy steel	621	7651	170
1.7131 (16 MnCr 5) high strength steel	880	7746	252
1.4541 (X6 CrNiTi 18 10) stainless steel	515	7521	166
Hardox 500 special low abrasion steel	1679	7524	544
3.1325 (duralumin 2017) duralumin	419	2784	122
2.0060 (E-Cu58) copper	211	8687	40
brass 2.0402 (CuZn40Pb2) brass	393	8364	61

**Table 3:** Samples from 34CrMo4 steel used for the production of pressure cylinders: austenitized, quenched and tempered.

Sample Mark (10 mm Thick)	Tempering Temperature (°C)	Uniaxial Strength (MPa)	Sample Mark (6 mm Thick)	Tempering Temperature (°C)	Uniaxial Strength (MPa)
K37	20	2230	K43	20	2160
K38	250	1865	K44	250	1860
K39	400	1560	K45	400	1550
K40	510	1340	K46	510	1320
K41	580	1190	K47	580	1230
K42	620	1060	K48	640	970

**Table 4:** The ratio of the x and z forces (cutting and deformation) measured by force sensor with different traverse speed sets (bold signed values), the respective limit traverse speeds are in a separate column and the relative traverse speeds are presented in brackets [ ].

**Sample Thickness (10 mm)**	**Limit *v_P_* Values**	**50 mm/min**	**100 mm/min**	**150 mm/min**
1.0547 (St 52-3)	194	**0.52** [0.26]	**0.71** [0.52]	**0.55** [0.77]
1.7131 (16 MnCr 5)	170	**0.49** [0.29]	**0.73** [0.59]	**0.54** [0.88]
1.0503 (C 45)	155	**0.56** [0.32]	**0.73** [0.65]	**0.45** [0.97]
1.4541 (X6 CrNiTi 18 10)	155	**0.53** [0.32]	**0.70** [0.65]	**0.48** [0.97]
**Sample Thickness (10 mm)**		**100 mm/min**	**150 mm/min**	**200 mm/min**
2.0060 (E-Cu 58)	254	**0.56** [0.39]	**0.62** [0.59]	**0.58** [0.79]
brass 2.0360 (CuZn40)	252	**0.62** [0.40]	**0.73** [0.60]	**0.55** [0.79]
**Sample Thickness (10 mm)**		**50 mm/min**	**75 mm/min**	**100 mm/min**
34CrMo4/* TT 20 °C	121	**0.50** [0.41]	**0.60** [0.62]	**0.51** [0.83]
34CrMo4/TT 250 °C	129	**0.60** [0.39]	**0.70** [0.58]	**0.61** [0.78]
34CrMo4/TT 400 °C	136	**0.60** [0.37]	**0.69** [0.55]	**0.57** [0.73]
34CrMo4/TT 510 °C	141	**0.50** [0.35]	**0.61** [0.53]	**0.52** [0.71]
34CrMo4/TT 580 °C	148	**0.52** [0.34]	**0.66** [0.51]	**0.58** [0.67]
34CrMo4/TT 620 °C	157	**0.50** [0.32]	**0.65** [0.48]	**0.59** [0.64]
**Sample Thickness (6 mm)**		**100 mm/min**	**125 mm/min**	**150 mm/min**
34CrMo4/TT 20 °C	186	**0.60** [0.53]	**0.68** [0.67]	**0.56** [0.81]
34CrMo4/TT 250 °C	204	**0.54** [0.48]	**0.67** [0.61]	**0.61** [0.73]
34CrMo4/TT 400 °C	221	**0.59** [0.45]	**0.66** [0.57]	**0.57** [0.68]
34CrMo4/TT 510 °C	227	**0.53** [0.44]	**0.67** [0.55]	**0.57** [0.66]
34CrMo4/TT 580 °C	228	**0.51** [0.43]	**0.65** [0.54]	**0.56** [0.65]
34CrMo4/TT 640 °C	253	**0.50** [0.40]	**0.60** [0.49]	**0.57** [0.59]

* TT—tempering temperature.

## Data Availability

No publicly archived datasets are reported or used.
